# Virtual Assessment of Physical Activity–Related Built Environment in Soweto, South Africa: What Is the Role of Contextual Familiarity?

**DOI:** 10.1007/s11524-024-00914-3

**Published:** 2024-09-10

**Authors:** Motlatso Godongwana, Khulu Gama, Vongani Maluleke, Lisa K. Micklesfield, Damilola Odekunle, Yves Florent Wasnyo, Boris Elouna, Edwin Ngwa, Adalberto Lopes, Muhammad Rabiu Balarabe, Eva Coringrato, Alice McGushin, Tolu Oni, Louise Foley, Tiago Canelas

**Affiliations:** 1 SAMRC-Wits Developmental Pathways for Health Research Unit, School of Clinical Medicine, Faculty of Health Sciences, University of the Witwatersrand, Johannesburg, South Africa; 2 Department of Urban and Regional Planning, University of Lagos, Lagos, Nigeria; 3 Centre for Housing and Sustainable Development, University of Lagos, Lagos, Nigeria; 4 Faculty of Medicine and Biomedical Sciences, University of Yaoundé 1, Yaoundé, Cameroon; 5 Health of Populations in Transition, University of Yaoundé 1, Yaoundé, Cameroon; 6 Faculty of Sciences, Department of Geography, University of Yaoundé 1, Yaoundé, Cameroon; 7 Observatory for Urban Health, Federal University of Minas Gerais, Belo Horizonte, Brazil; 8 African Centre for Cities, University of Cape Town, Cape Town, South Africa; 9 RAND Europe, Cambridge, UK; 10 MRC Epidemiology Unit, University of Cambridge, Cambridge, UK; 11 Burden of Disease Research Unit, South Africa Medical Research Council, Tygerberg, South Africa

**Keywords:** Built environment, Physical activity, Contextual familiarity, MAPS-Global, Virtual assessment

## Abstract

Understanding how urban environments shape physical activity is critical in rapidly urbanizing countries such as South Africa. We assessed the reliability of virtual audits for characterizing urban features related to physical activity in Soweto, South Africa. We used the Microscale Audit of Pedestrian Streetscapes Global tool to characterize pedestrian-related features from Google Street View images in four neighborhoods of Soweto. Neighborhoods were selected to represent different levels of deprivation. Inter-rater reliability was analyzed according to the rater’s familiarity with the local area. The results show a higher inter-rater reliability was observed among auditors with greater contextual familiarity. Many measurements however generated inconclusive results due to either low variability in the raters’ responses or the absence of the features in the streets. It is evident from our findings that virtual audits are efficient tools that can be used to assess the built environment. However, to ensure meaningful use of these tools in diverse settings, we recommend that auditors comprise of people with contextual familiarity.

**Supplementary Information:**

The online version contains supplementary material available at 10.1007/s11524-024-00914-3.

## Introduction

By 2050, the majority of urban growth is expected to occur in low-income and middle-income countries (LMICs) [1]. South Africa exemplifies this trend, experiencing dramatic urban expansion in recent decades [2]. This pivotal moment of expansion, transformation in urban planning, and informal urban development directly influences the built environment, which is closely linked to residents’ physical activity levels [3, 4]. Understanding these dynamics is crucial for developing cities that promote healthier lifestyles [5].

Globally, one in four adults and four in five adolescents are insufficiently physically active despite the substantial health benefits of physical activity [6, 7]. It was estimated that only 60% of South Africans are meeting the World Health Organization (WHO) recommended standards for PA [8]. Despite the WHO targets to reduce physical inactivity by 15% by 2030 [9] and the ambitious policies to create healthy cities that will increase physical activity, several studies have found that there is a gap between what has been implemented and what is needed to achieve these targets [4, 5]. However, lack of data regarding the built environment in LMICs cities is a barrier for creating healthy environments that support physical activity [10].

Traditionally, the collection of the built environment data has been carried out through field audits whereby assessors walk a predetermined route through a specific area and use an observational form to assess predefined environmental characteristics [10, 11]. The Microscale Audit of Pedestrian Streetscapes Global version (MAPS-Global) is one of the tools used to measure and characterize features of the built environment such as street characteristics, sidewalks, intersections, streets aesthetics, and other design features which may help to explain physical activity variation within a population [12]. However, where these assessments are most relevant and needed, such as highly urbanized African cities, very little evidence exists on the nature of the built environment. Assessing the built environment as a first step is important for measuring the association between features of the built environment and physical activity.

The use of virtual assessment tools has been advocated to reduce the time and resources required for conducting in-person audits [11, 13, 14]. In high-income countries, these tools have been found to be reliable assessments of the built environment [11, 13, 15, 16]. Few researchers have explored the concept of contextual familiarity (living or working in the study area vs outside the study area) when assessing the reliability of the tools [13, 16, 17]. This concept of familiarity becomes paramount when conclusions about the reliability of virtual tools are drawn from studies that do not contemplate African settings [10, 18]. The African setting is unique because of its informality and factors including rapid urbanization. Thus, the aim of this study was to measure the reliability among raters with different levels of familiarity to a highly urbanized African city using the MAPS-Global tool.

## Methods

We conducted virtual audits of the built environment in Soweto, South Africa. Soweto is located in Johannesburg and was established in 1931 as a result of spatial segregation laws during the apartheid regime in South Africa. The region is now an urban settlement characterized by varying levels of socioeconomic deprivation with a population of approximately 1.9 million people living in a 200 km^2^ area [19].

**Fig. 1:**
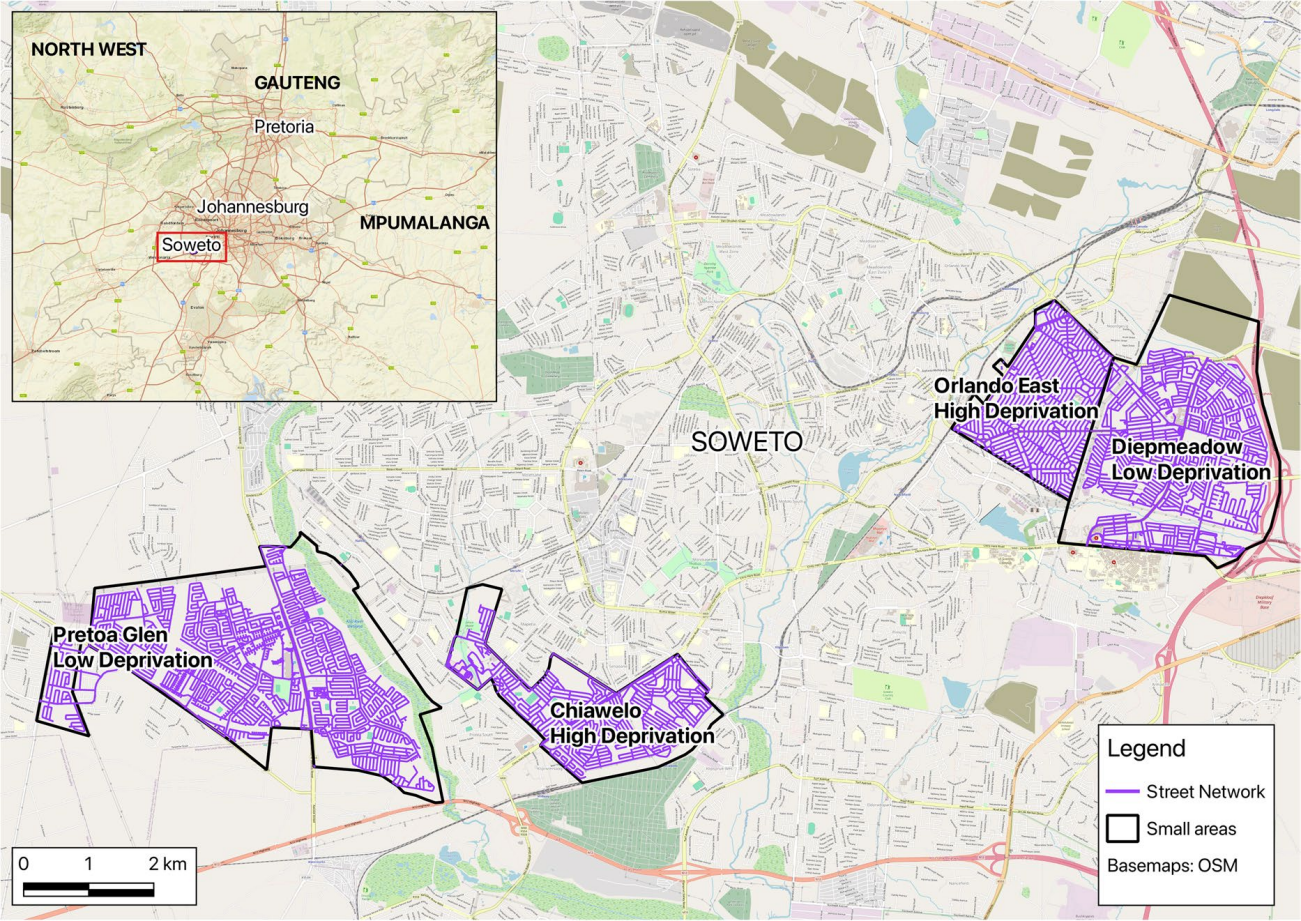
Reference map of the small areas audited and their deprivation level

We collected data from four small areas within Soweto: Chiawelo, Diepmeadow, Orlando East, and Protea Glen (Fig. 1). We purposively selected the areas to provide variation in socioeconomic deprivation (two areas of higher deprivation, two of lower deprivation). We determined deprivation level based on a methodology previously outlined by The Healthy Life Trajectories Initiative (HeLTi) enumeration study that collected data in Soweto and local knowledge from the study team. In this study, Protea Glen and Diepkloof were assigned as areas of higher SES (low deprivation) and Chiawelo and Orlando East as lower SES (high deprivation) [20].

To assess features of the built environment, we used MAPS-Global [12, 13]. The MAPS-Global tool was developed by researchers from the University of California San Diego and validated across countries with varying built environmental characteristics such as Australia, Belgium, Brazil, China, and Spain [21]. The instrument comprises four sections, collected along a predefined route: (1) segment (measures block faces between intersections); (2) crossings (collects information on street intersections); (3) route (evaluates destinations and use, streetscape characteristics and aesthetic and social characteristics from a defined origin to a defined destination); (4) cul-de-sac (assesses amenities in dead ends).

For this study, the routes were chosen using a geographically stratified sampling design. Specifically, the selection process involved three key features: firstly, random households (extracted from the OpenBuildings dataset [22]) were utilized as starting points; secondly, local points of interest (POIs) identified by the local team served as endpoints; and thirdly, the street network (from OpenStreetMaps [23]) functioned as the connecting routes between these starting and ending points. Routes had a length between 400 and 700 m. For each small area, sampled routes covered 25% of the total street network, which was considered to give an adequate representation of the built environment in that small area [24]. Data collection was conducted using the Google Street View (GSV) functionality within Google Earth Pro, where the designated routes were uploaded.

The virtual audits were conducted between April and May 2023, and data collection took place in two phases. The auditing team consisted of 10 researchers collaborating with the Global Diet and Physical Activity (GDAR) network from five different countries (South Africa, Nigeria, Cameroon, the United States, and the UK). There were three categories of auditors, seven with no experience of the Soweto context (none), three who worked in the Soweto area (context), and two auditors from Soweto who had conducted field audits on the same streets 9 months prior to the virtual assessment (field). The two field auditors are also within the context group.

All auditors participated in an online training session to standardize the data collection methodology using the MAPS-Global material [25]. Subsequently, each auditor was tasked to assess seven routes in phase one, and three routes in phase two. All auditors assessed the same set of routes.

Data entry was completed in REDCap, both via its online platform and mobile application. REDCap’s functionality also enabled the upload of precise counts of segments and crossings (which varied by route), which were required for conducting the intraclass correlation coefficient (ICC) analysis (using R version 3.x). This study expands on the preliminary GDAR research assessing the built and food environments in four African cities (unpublished data), for which five items from MAPS-Global were incorporated into a different assessment of the food environment. Therefore, these five items were not scored in the current study. The list of items used in the current study can be found in Supplementary Table 1.

The inter-rater reliability of MAPS-Global was measured on several single-item indicators, subscales, valence scores (composite of positive or negative), and overall scores as described in Millstein et al. [26]. Numerical data was assessed with the ICC measurement and Cohen’s kappa coefficients for categorical data using the package “*pysch*” in R version 3.x. For this study, the ICC and Cohen’s kappa were classified to indicate inter-rater reliability that was “excellent” (ICC ≥ 0.75), “good” (0.60–0.74), “fair” (0.40–0.59), and “poor” (< 0.40) [27]. If the absence of features in the subscales, valence, and overall scores creation was higher than 80% (i.e., places of worship, private recreational facilities, etc.), we excluded it from the analysis as there would not be enough variability for a correct interpretation of the ICC.

## Results

### The Feasibility and Operational Practicality of Virtual Assessments in Soweto

We encountered two significant challenges in adhering to the MAPS-Global auditing procedures in phase 1 of data collection. Firstly, the coverage of several areas by GSV was incomplete, with GSV coverage for routes as low as 14.3%, limiting our ability to complete the audits in some streets. Secondly, inconsistencies in the number of segments and crossings recorded by different auditors hindered our ability to make comparisons. These challenges contributed to fluctuations in assessment times and inter-rater reliability.

To address these issues in phase 2 of the data collection, we excluded routes with less than 75% GSV coverage. The challenge of discrepancies in the number of segments and crossings was partly derived from Soweto’s street layouts, which differ from those assumed in MAPS-Global procedures. For example, some streets lack sidewalks, complicating the determination of safe pedestrian passages and crossing points. Acknowledging this limitation, the trainers standardized the number of segments and crossings for each route prior to audit, enabling consistent comparisons between auditors.

In addition to the initial challenges, we also encountered significant network issues, as the auditors’ limited broadband access delayed the auditing process. During the debriefing following the second phase of data collection, some auditors revealed they used Google searches to confirm or identify elements that were difficult to discern in the GSV images, particularly concerning land use, such as the presence of amenities along the route or the type of business present.

In the second phase, a total of three routes, 19 segments, and 16 crossings were analyzed virtually by all auditors. Most of the images used were approximately 1 year old, although some were as old as 10 years. The second phase of data collection showed marked improvement from the first phase, with GSV coverage rates reaching almost full coverage (96% vs. 76% in the first phase) for all routes. The data collection process was significantly more efficient, with the mean assessment time for routes at 7.7 ± 6 min (compared with 12.3 ± 11.6 min in the first phase). Furthermore, the average time to assess segments and crossings was reduced to 4.1 ± 2.2 and 1.1 ± 0.1 min, respectively (compared with 4.9 ± 6.3 min and 2 ± 2.6 min in the first phase) indicating a more consistent and streamlined auditing procedure. Data entries where image date coincided with the collection day were excluded from the analysis as they were considered a methodological error by auditors. Cul-de-sacs were not included in the analysis as there was none on the routes audited. Detailed descriptions of each route are delineated in Supplementary Table 2.

### The Influence of Familiarity in the IRR

**Fig. 2:**
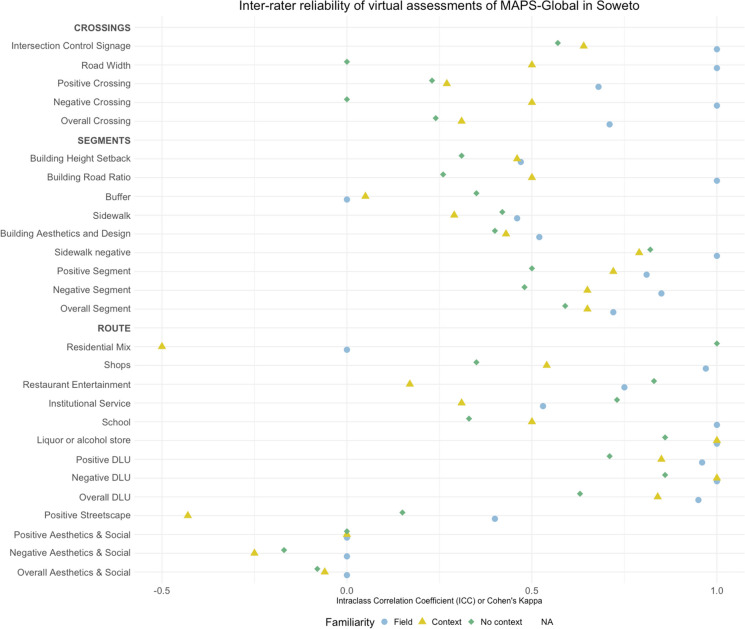
Inter-rater reliability for virtual MAPS-Global assessments in Soweto

Overall, we found that contextual familiarity was associated with greater inter-rater reliability of virtual audits in Soweto. Figure 2 shows that for almost all the subscales, valence, and overall scores, inter-rater reliability was higher when the online auditors were familiar with the context. We calculated measurements for only 30 out of 41 items or subscales, adhering to the criterion that required more than 80% presence for calculation.

### Routes

Detailed results for route reliability subscales and valence scores are presented in Table 1. In overall, for the route section, reliability was markedly lower compared with segment and crossing, with no clear pattern by familiarity.

**Table 1:** MAPS-Global—route section item-level and subscale inter-rater reliability and descriptive statistics

Route section—variable description	# items (range of scores)	Null count (%)	Mean (S.D)	ICC/Kappa CI (95%)	Sample items and overall subscale description
**Positive destinations and land use**					
Institutional-service	3 (0–15)	12.5%	f: 4.33 (2.25)	0.53 (− 0.85 to 0.98)	Bank, health-related professional, other service
			c: 4 (1.87)	0.31 (− 0.35 to 0.97)	
			n: 2.73 (2.05)	0.73 (0.21–0.99)	
Private recreation	2 (0–10)	100%	f: 0	NA	Private indoor, private outdoor facility
			c: 0		
			n: 0		
Public recreation	4 (0–20)	83.3%	f: 0.17 (0.41)	NA	Public indoor, public outdoor facility, park, trail
			c: 0.22 (0.44)		
			n: 0.13 (0.35)		
Residential mix	4 (0–3)	0.0%	f: 1.5 (0.55)	0*	Single family, multi-family, mixed, apartment over retail
			c: 1.33 (0.5)	− 0.5*	
			n: 1 (0)	1.00*	
Restaurant-entertainment	4 (0–20)	16.7%	f: 2.67 (1.63)	0.75 (− 0.70 to 0.99)	Fast food, sit-down, café, entertainment
			c: 2.67 (2.65)	0.17 (− 0.39 to 0.95)	
			n: 2.27 (1.75)	0.83 (0.39–1.00)	
School	1 (0–5)	75%	f: 0.33 (0.52)	1.00 (1.00–1.00)	School land use
			c: 0.22 (0.44)	0.50 (− 0.18 to 0.98)	
			n: 0.27 (046)	0.33 (− 0.09 to 0.96)	
Shops	8 (0–28)	0.0%	f: 4.83 (2.04)	0.97 (0.22–1.00)	Grocery, convenience store, bakery, drugstore, other retail, shopping mall, strip mall, open-air market
			c: 5.11 (1.83)	0.54 (− 0.24 to 0.98)	
			n: 4.27 (2.09)	0.35 (− 0.09 to 0.97)	
Worship	1 (0–5)	100%	f: 0	NA	Place of worship
			c: 0		
			n: 0		
**Negative destinations and land use**					
Age-restricted bar or nightclub	1 (0–5)	100%	f: 0	NA	Age-restricted bar or nightclub
			c: 0		
			n: 0		
Liquor or alcohol store	1 (0–5)	33.3%	f: 0.67 (0.52)	1.00 (1.00–1.00)	Liquor or alcohol store
			c: 0.67 (0.50)	1.00 (1.00–1.00)	
			n: 0.73 (0.59)	0.86 (0.50–1.00)	
**Valence and overall scores**					
Positive DLU	28 (0–111)	0.0%	f: 11.9 (5.12)	0.90 (− 0.33 to 1.00)	Sum of the positive DLU subscales
			c: 15.2 (3.93)	0.85 (0.20–1.00)	
			n: 15.7 (3.20)	0.72 (0.21 – 0.99)	
Negative DLU	2 (0–10)	33.3%	f: 0.67 (0.52)	1.00 (1.00–1.00)	Sum of the negative DLU subscales
			c: 0.67 (0.50)	1.00 (1.00–1.00)	
			n: 0.73 (0.59)	0.86 (0.50–1.00)	
Overall DLU	30	0.0%	f: 15 (2.97)	0.88 (− 0.42 to 1.00)	Positive DLU—negative DLU
			c: 14.6 (3.75)	0.83 (0.33–1.00)	
			n: 11.2 (4.74)	0.64 (0.11–0.99)	
**Streetscape characteristics**					
Positive streetscape	25 (0–22)	20.8%	f: 2.83 (2.86)	0.40 (− 0.89–0.98)	Transit, traffic calming, trash bins, benches, bike racks, bike lockers, bike docking stations, kiosks, hawkers
			c: 2.67 (2.55)	− 0.43 (− 0.49 to 0.04)	
			n: 2.07 (1.87)	0.15 (− 0.16 to 0.94)	
**Aesthetics and social characteristics**					
Positive aesthetics / social	4 (0–4)	54.2%	f: 1 (1.26)	0 (− 0.95 to 0.95)	Hardscape, water, softscape, landscaping
			c: 0.67 (1.12)	0 (− 0.43 to 0.93)	
			n: 0.60 (0.63)	0 (− 0.2 to 0.88)	
Negative aesthetics / social	6 (0–5)	8.3%	f: 0.67 (0.52)	0 (− 0.95 to 0.95)	Buildings not maintained, graffiti, litter, dog fouling, physical disorder, highway near
			c: 0.89 (0.60)	− 0.25 (− 0.47 to 0.83)	
			n: 1.33 (0.49)	− 0.17 (− 0.21 to 0.64)	
Overall aesthetics / social	10	25%	f: 0.33 (1.75)	0 (− 0.95 to 0.95)	Positive aesthetics/social—negative aesthetics/social
			c: − 0.22 (1.64)	− 0.06 (− 0.44 to 0.91)	
			n: − 0.73 (0.88)	− 0.08 (− 0.22 to 0.83)	

Familiarity: *f*: field, *c*: context, *n*: none. Agreement: “excellent” (ICC ≥ 0.75), “good” (0.60–0.74), “fair” (0.40–0.59), and “poor” (< 0.40). *DLU*, destinations and land use; *ICC*, intraclass correlation coefficient; *CI*, confidence interval; *SD*, standard deviation; *NA*, not applicable. *Indicates the analysis was Cohen’s Kappa

#### Destinations and Land Use

We evaluated five out of eight positive subscales and single items, because three (place of worship, public and private recreation) had over 80% zeros. Interestingly, in the destinations and land use section, unfamiliarity with the local context was associated with higher reliability, in contrast with the valence and overall scores. Notably high agreement between auditors who were not familiar with the context was observed in the assessment of residential mix (ICC = 1.00, 95% CI [1.00, 1.00]), and restaurants and entertainment (ICC = 0.83, 95% CI [0.39, 1.00]), and institutional services (ICC = 0.73, 95% CI [0.21, 0.99]). Conversely, agreement on the numbers of schools (ICC = 1.00, 95% CI [1.00, 1.00]) and shops (ICC = 0.97, 95% CI [0.22, 1.00]) was higher in field-experienced auditors compared to the other familiarity groups. Among negative subscales, none of the raters identified any age-restricted bar or nightclub. The presence of liquor or alcohol stores yielded near-perfect agreement between all familiarity groups, with the field and context team achieving a perfect score. Upon measuring positive and negative valences along with the overall scores, the influence of familiarity became evident, as the field group exhibited higher ICC values than the other groups.

#### Streetscape Characteristics

The streetscape’s positive subscale revealed no cycling infrastructure across audited routes. Notably, the mean count for all familiarity groups was below three streetscape features per route (out of a maximum of 22) noting a very low presence of amenities in the surveyed routes. The ICC for all familiarity groups was poor, or with negative values, implying that any agreement among raters was lower than what would be expected by chance alone.

#### Aesthetics and Social

Both the positive and negative subscales, as well as the overall aesthetics and social scale, demonstrated a lack of consensus among auditors, unaffected by their familiarity with the area. Notably, no measurements exceeded 0, suggesting either random variations in ratings or lower agreement than by chance.

### Segments

Detailed results for segment reliability subscales, valence, and overall scores are presented in Table 2. Two categories, cycling infrastructure and informal path or shortcut, had over 80% zeros, indicating a lack of these features in the audited areas. Familiarity with the local context variably influenced agreement levels for the different segment subscales. The field group showed higher agreement between the auditors in both the positive (ICC = 0.85, 95% CI [0.64, 0.94]) and negative (ICC = 0.85, 95% CI [0.65, 0.94]) valence scores, as well as the overall score. All the subscales with exception of the buffer had the field or context group achieving the highest reliability.

**Table 2:** MAPS-Global—route section item-level and subscale inter-rater reliability and descriptive statistics

Segments section—variable description	# items (range of scores)	Null count (%)	Mean (S.D)	ICC/Kappa CI (95%)	Sample items and overall subscale description
**Positive segment subscales**					
Bicycle infrastructure	3 (0–15)	100%	f: 0	NA	Bank, health-related professional, other service
			c: 0		
			n: 0		
Buffer	2 (0–5)	46.10%	f: 1.95 (0.32)	0 (− 0.44 to 0.44)	Parking along street, buffer
			c: 1.95 (0.32)	0.05 (− 0.18 to 0.37)	
			n: 0.96 (1.41)	0.35 (0.16–0.60)	
Building aesthetics and design	1 (0–2)	41.30%	f: 1.63 (0.75)	0.52 (0.10–0.78)	Street windows
			c: 1.39 (0.86)	0.43 (0.15–0.70)	
			n: 0.63 (0.73)	0.40 (0.19–0.64)	
Building height-road width ratio	5 (0–3)	4.70%	f: 2.81 (0.7)	1.00 (1.00–1.00)	Building height, setback and road width
			c: 2.87 (0.58)	0.50 (0.23–0.74)	
			n: 2.45 (0.86)	0.26 (0.08–0.52)	
Building height-setback	4 (0–10)	4.61%	f: 3.34 (1.36)	0.47 (0.03–0.75)	Building height, smallest and largest setback
			c: 3.74 (1.41)	0.46 (0.18–0.72)	
			n: 3.41 (1.82)	0.31 (0.18–0.72)	
Hawkers/shops	1 (0–2)	71.60%	f: 0.27 (0.45)	0.23 (− 0.24 to 0.61)*	Hawkers/shops on sidewalk/pedestrian zone
			c: 0.43 (0.57)	0.31 (0.03–0.61)*	
			n: 0.22 (0.42)	0.10 (− 0.04 to 0.34)*	
Informal path or shortcut	1 (0–1)	85.20%	f: 0.13 (0.34)	NA	Informal path connecting to something else
			c: 0.11 (0.31)		
			n: 0.17 (0.38)		
Pedestrian infrastructure	5 (0–5)	44.70%	f: 0.42 (0.55)	0.48 (0.05–0.76)	Mid-segment crossing, pedestrian bridge, covered place to walk, street lights
			c: 0.42 (0.53)	0.63 (0.38–0.82)	
			n: 0.67 (0.53)	0.40 (0.19–0.64)	
Shade	3 (0–6)	36.80%	f: 0.61 (0.64)	0.56 (0.16–0.80)	Number of trees, sidewalk coverage, shade
			c: 0.63 (0.62)	0.59 (0.34–0.80)	
			n: 0.74 (0.64)	0.58 (0.38–0.78)	
Sidewalk	2 (0–6)	2.63%	f: 4.18 (0.98)	0.46 (0.03–0.75)	Sidewalk presence and width
			c: 3.98 (1.17)	0.29 (0.02–0.60)	
			n: 3.88 (0.94)	0.42 (0.22–0.66)	
**Valence and overall scores**					
Positive segment	27 (0–45)	0.00%	f: 15.3 (2.97)	0.85 (0.64–0.94)	Sum of the positive segment subscales
			c: 14.9 (2.99)	0.75 (0.54–0.88)	
			n: 13.1 (4.14)	0.51 (0.31–0.73)	
Negative segment	7 (0–13)	0.00%	f: 5.24 (1.82)	0.85 (0.65–0.94)	Sum of the negative segment single items (non-continuous sidewalk, trip hazards, obstructions, cars blocking walkway, slope, gates, driveways)
			c: 5.09 (1.71)	0.65 (0.41–0.83)	
			n: 4.2 (1.43)	0.48 (0.27–0.66)	
Overall segment	34	0.00%	f: 10 (3.05)	0.76 (0.48–0.90)	Positive segment—negative segment
			c: 9.84 (3.17)	0.67 (0.44–0.84)	
			n: 8.89 (4.3)	0.59 (0.39–0.78)	

Familiarity: *f*: field, *c*: context, *n*: none. Agreement: “excellent” (ICC ≥ 0.75), “good” (0.60–0.74), “fair” (0.40–0.59), and “poor”’ (< 0.40). *DLU*, destinations and land use; *ICC*, intraclass correlation coefficient; *CI*, confidence interval; *SD*, standard deviation; *NA*, not applicable. *Indicates the analysis was Cohen’s Kappa

### Crossings

Detailed results for crossing reliability subscales, valence, and overall scores are presented in Table 3. The only positive crossing subscale that did not have more than 80% zeros was the intersection control and signage subscale and this still only reported the mean number of features as < 1/ crossing. Notably, crosswalk amenities, which are crucial for safe road crossing, had 90% zeros. In the negative subscale assessing road width, the group unfamiliar with the context showed no agreement, while those with context knowledge scored fair to excellent agreement. In the overall score, only the field auditors reached an excellent score and the other groups a fair reliability.

**Table 3:** MAPS-Global—route section item-level and subscale inter-rater reliability and descriptive statistics

Crossing section—variable description	# items (range of scores)	Null count (%)	Mean (S.D)	ICC/Kappa CI (95%)	Sample items and overall subscale description
**Positive crossing subscales**					
Bicycle feature	3 (0–3)	99.2%	f: 0	NA	Waiting area, bike lane crossing the crossing, bike signal
			c: 0.01		
			n: 0		
Crosswalk amenities	7 (0–7)	90.6%	f: 0	NA	Crossing aids, marked crosswalk, high visibility striping, different material, curb extension, raised crosswalk, refuge islands
			c: 0.09 (0.33)		
			n: 0.13 (0.33)		
Curb quality and presence	3 (0–5)	88.30%	f: 0.13 (0.55)	NA	Curb presence, curb ramps lined up, tactile paving
			c: 0.34 (1.11)		
			n: 0.42 (1.11)		
Intersection control and signage	7 (0–8)	39.80%	f: 0.63 (0.49)	1.00 (1.00–1.00)	Yield signs, stop signs, traffic signal, traffic circle, pedestrian walk signals, push buttons, countdown signal
			c: 0.61 (0.53)	0.64 (0.37–0.84)	
			n: 0.63 (0.49)	0.57 (0.35–0.79)	
Overpass	1 (0–1)	100%	f: 0	NA	Crossing on pedestrian overpass, bridge
			c: 0		
			n: 0		
**Negative crossing subscales**					
Road width	1 (0–2)	60.90%	f: 0.06 (0.25)	1.00 (1.00–1.00)	Distance of crossing leg
			c: 0.61 (0.52)	0.50 (0.20–0.76)	
			n: 0.04 (0.20)	0.00 (− 0.12 to 0.23)	
**Valence and overall scores**					
Positive crossing	21 (0–24)	39.80%	f: 0.63 (0.49)	1.00 (1.00–1.00)	Sum of the positive crossing subscales
			c: 0.61 (0.53)	0.64 (0.37–0.84)	
			n: 0.63 (0.49)	0.57 (0.35–0.79)	
Negative crossing	1 (0–2)	60.90%	f: 0.06 (0.25)	1.00 (1.00–1.00)	Sum of the negative crossing subscales
			c: 0.61 (0.52)	0.50 (0.20–0.76)	
			n: 0.04 (0.20)	0 (− 0.12 to 0.23)	
Overall	22	39.80%	f: 0.56 (0.5)	1.00 (1.00–1.00)	Positive crossing—negative crossing
			c: 0.58 (0.5)	0.58 (0.28–0.81)	
			n: 0.0 (0.68)	0.55 (0.32–0.77)	

Familiarity: *f*: field, *c*: context, *n*: none. Agreement: “excellent” (ICC ≥ 0.75), “good” (0.60–0.74), “fair” (0.40–0.59), and “poor” (< 0.40). *DLU*, destinations and land use; *ICC*, intraclass correlation coefficient; *CI*, confidence interval; *SD*, standard deviation; *NA*, not applicable. *Indicates the analysis was Cohen’s Kappa

#### Grand Score Reliability

Detailed results for the positive, negative, and final overall score are presented in Table 4. Across all three scales, we observe a familiarity gradient, with the field group having higher reliability than the other groups. However, the mean scores among rater familiarity groups are notably similar, indicating that the presence or degree of context familiarity does not markedly distinguish these groups.

**Table 4:** MAPS-Global—route section item-level and subscale inter-rater reliability and descriptive statistics

Overall section—variable description	# items (range of scores)	Null count (%)	Mean (S.D)	ICC/Kappa CI (95%)	Sample items and overall subscale description
Overall positive	101 (0–205)	0.0%	f: 35 (3.63)	0.95 (0.02–1.00)	Positive DLU, positive streetscape, positive aesthetics/social, positive segment (mean of all segments), positive crossing (mean of all segments)
			c: 33.6 (4.41)	0.67 (− 0.12 to 0.99)	
			n: 27.8 (7.85)	0.83 (0.39–1.00)	
Overall negative	16 (0–22)	0.0%	f: 7.01 (2.09)	0.98 (0.36–1.00)	Negative DLU, negative aesthetics/social, negative segment (mean of all segments), negative crossing (mean of all crossings)
			c: 6.96 (1.66)	0.52 (− 0.25 to 0.98)	
			n: 7.03 (0.95)	0.76 (0.26–0.99)	
Overall	113	0.0%	f: 28 (4.28)	0.95 (0.00–1.00)	Overall Positive – Overall Negative
			c: 26.7 (4.71)	0.89 (0.31–1.00)	
			n: 20.8 (7.96)	0.84 (0.41–1.00)	

Familiarity: *f*: field, *c*: context, *n*: none. Agreement: “excellent” (ICC ≥ 0.75), “good” (0.60–0.74), “fair” (0.40–0.59), and “poor” (< 0.40). *DLU*, destinations and land use; *ICC*, intraclass correlation coefficient; *CI*, confidence interval; *SD*, standard deviation; *NA*, not applicable. *Indicates the analysis was Cohen’s Kappa

## Discussion

To our knowledge, this is the first study assessing the inter-rater reliability of MAPS-Global in an African urban context, and our findings highlight the importance of local knowledge in applying research tools effectively. Our findings suggest that auditors with local familiarity yielded more reliable audits compared to their international peers. Despite the global accessibility of virtual platforms like Google Street View and Google Earth for environmental assessment, our results underscore the value of contextual familiarity in enhancing the meaningful application and rigor of research tools.

Incorporating contextual familiarity in global health research is crucial and at the same time an ethical responsibility when the tools we use have not been validated in the contexts where we work [28]. This practice risks oversimplifying complex realities and may lead to wrong or misleading conclusions. Rzotkiewicz et al. (2018) highlighted this gap, noting the absence of studies using Google Street View in Africa and limited research in Latin America and Asia, challenging the assumption of universal applicability for virtual audits.

This study contributes from an African setting to the limited and inconclusive research on rater familiarity in evaluating the built environment. Two studies, one utilizing the MAPS-Global in Belgium and the other applying S-VAT tool in Norway, demonstrated that auditors with greater contextual familiarity or those conducting audits in-person reported higher inter-rater reliability [15, 17]. On the other hand, Fox et al. (2021) using MAPS-Global in five HICs countries and Zhu et al. (2017) using MAPS-Global in the US suggested that familiarity does not significantly affect virtual audit outcomes. Most studies that have used virtual tools to characterize the built environment have been carried out in HICs with different environmental characteristics compared to LMICs [13, 15–17, 29, 30]. Their studies present findings from well-planned cities making it difficult to draw similar conclusions to a highly urbanized and dynamic environment of the Soweto township.

Not only are LMICs underrepresented in virtual auditing, but there are also acute spatial inequalities in the amount of GSV coverage within cities depending on deprivation levels [31]. Several authors have stated that virtual audits are a reliable alternative to in-person street audits but with a caveat that there is the need for high coverage and updated images [13, 17]. Our study tackled the variability in coverage by selecting routes with at least 75% visibility. However, assessing the recency of GSV images posed a challenge, as image dates can vary widely even within the same location, depending on the viewing angle. Incorporating insights from a local team regarding acceptable image year ranges can significantly enhance the relevance of urban assessments, especially in LMICs where rapid urbanization is prevalent (Ritchie et al., 2024; UN-Habitat, 2022). This dialogue with the local auditors is crucial due to the continuous and fast-paced urban changes, underscoring the necessity for up-to-date imagery in environmental audits of the built environment.

Our auditors faced several challenges, including issues with image quality, outdated images, blurriness, and obstructions, similar to studies elsewhere [13, 15, 18]. An unexpected challenge emerged from feedback sessions: some resorted to using search engines to identify unclear elements in images. This practice potentially introduced inconsistencies in the auditing process, emphasizing the need for clearer guidelines in the training to ensure uniformity in virtual environmental assessments [13, 32, 33]. Although it is not unusual to find a high percentage of absence in some features of the microscale [11, 13], we found our study to lack many of the features of MAPS-Global. This highlights the lack of many essential amenities in these low-resourced settings but also raises the concern whether MAPS-Global was indeed the correct tool for our study site. The choice of MAPS-Global was made collectively by the GDAR Network members [34]. To enhance representativity, future studies using global audit tools should consider the differences within and between neighborhoods, regions, and countries. It is important to note that developmental patterns, urbanization levels, land uses, and socioeconomic statuses of residents have different definitions, interpretations, and representations across the world.

Similar to other virtual audits of the built environment, the subjective features of the built environment such as the streets or building aesthetics had the lowest IRR across all the MAPS-Global measurements [13, 15, 16, 33]. Our findings indicated inter-rater reliability was highest in the land use section of the routes, similar to results from Zhu et al. (2017). The high frequency of null responses in the crossing section (indicating a lack of infrastructure to facilitate road crossing) is notable in a country where pedestrians constitute almost 40% of road traffic fatalities [35]. Additionally, a study in a low-income community in South Africa showed that half of the children walking to school alone report experiences with pedestrian collisions [36].

We acknowledge the limitations of our study posed by a small sample size, due to a combination of challenges, such as image coverage and limited time resources. While certain measurements, such as the pedestrian buffers, showed high percentage of agreement across all auditors, the statistical measures of reliability, such as ICC or Kappa, indicated lower values. This discrepancy stems from limited variability in exposure, where we obtained low ICC or Kappa despite a high percent agreement [16, 37]. However, the adoption of virtual auditing markedly decreased the costs associated with conducting audits, offering a more economical alternative to traditional in-field methods. The auditors categorized as field familiarity for this study conducted in-field audits in the same area nine months prior, and the virtual audits were completed faster (unpublished data). Additionally, while in-field audits necessitated pairs of auditors for safety reasons, virtual audits allowed individuals to work solo, providing flexibility and the comfort of conducting audits from any location.

## Conclusion

We advocate for the thoughtful application of virtual audits in highly urbanized African cities. Utilizing raters familiar with the local context will help to ensure the benefits of virtual audits, including efficiency, resource allocation, and safety, are realized. However, key factors need to be considered including image coverage, the recency of images, dynamic, and the suitability of global tools to capture local environments. Careful evaluation of these aspects would ensure that auditors are well placed to conduct accurate and effective virtual audits.

## Supplementary Information

Below is the link to the electronic supplementary material.

Supplementary file1 (DOCX 21 KB)

Supplementary file2 (DOCX 17 KB)

## Data Availability

The datasets during and/or analyzed during the current study are available from the corresponding author on reasonable request.
