# Supplementary material for: Virtual Assessment of Physical Activity–Related Built Environment in Soweto, South Africa: What Is the Role of Contextual Familiarity?
Source: J Urban Health. 2024 Sep 10;101(6):1221–34. doi: 10.1007/s11524-024-00914-3 (PMC11652452; doi:10.1007/s11524-024-00914-3)
Supplement: Supplementary file 1 — Supplementary file1 (DOCX 21 KB) [file 11524_2024_914_MOESM1_ESM.docx]

**Supplementary table 1:** List of all the questions.

| **ROUTES** | **SEGMENTS** | **CROSSINGS** |
| --- | --- | --- |
| Start date | Start date | Start date |
| Start time | Start time | Start time |
| Is ALL the route accessible through Google Street View? | Year of the Google Street View image | Year of the Google Street View image |
| Percentage of Google Street View images available in this route | Is it a paved road? | Percentage of Google Street View images available in this crossing |
| What types of residential uses are in the route? | How many traffic lanes are present in your side of the segment? | Intersection control |
| Fast food restaurants | What % of the segment is parking allowed (formal or informal)? | Does this crossing take place on a pedestrian overpass, underpass or bridge? |
| Sit down restaurants or bar (all-ages) | Is a sidewalk present? | Pedestrian signalization |
| Supermarket / hypermarket | What is the width of the majority of the sidewalk? | Pre-crossing curb (ramps) |
| Convenience/general store | Is there a buffer present? | Post-crossing curb (ramps) |
| Cafes/fast casual restaurants | Are there poorly maintained sections of the sidewalk that constitute major trip hazard? | Is tactile paving provided at curbs? |
| Bakery | Are there hawkers or shops on the sidewalk or pedestrian street/zone? | Are there crossing aids present? |
| Age-restricted bar/nightclub | Are there signs, bus shelters, kiosks and street furniture obstructing the sidewalk or pedestrian street/zone? | Crosswalk treatment |
| Alcohol specialty stores | Are there cars blocking the sidewalk or pedestrian street/zone? | Is a protected refuge island present? |
| Cash in - cash out (bank/credit union/ATM/Phone money/Western Union etc.) | Is there an informal path (shortcut) which connects to something else? | Count how many lanes are in this crossing, including all traffic and turn lanes |
| Drugstore/pharmacy (official or no official) | What is the slope of the majority of the segment? | Is a waiting area (bike box) provided for cyclists that stop at the crossing? |
| Public nurse-only clinic | How many trees exist within 1.5m of either side of the sidewalk/pathway? | Does a bike lane or path cross the crossing? |
| Private nurse-only clinic | What % of the length of the sidewalk/walkway is covered by trees? | Finish time |
| Public medical clinic | What % of the length of the sidewalk/walkway is covered by awnings or other overhead coverage? |  |
| Private medical clinic | What is the smallest building setback from the sidewalk? |  |
| Public hospital | What is the larger setback from the sidewalk/walkway? |  |
| Private hospital | What is the shortest building height? |  |
| Traditional health provider | What is the tallest building height? |  |
| Entertainment | How many properties are protected by gates, walls or tall fences (>2m height)? |  |
| Other services | How many driveways are there? |  |
| Other retail | Estimate the proportion of street segment that has ground floor or street-level windows within 12m of sidewalk/walkway |  |
| Place of worship | Is there a pedestrian mid-segment crossing? |  |
| Pre-school, Primary or Secondary School | Is it a pedestrian bridge/overpass or tunnel? |  |
| Other schools | Is there a covered or air conditioned place to walk along the street or connecting buildings (not a mall)? |  |
| College/University/Post-secondary Technical college | Is there a bicycle lane or zone |  |
| Private indoor recreation | What is the quality of the bicycle lane or zone? |  |
| Public indoor recreation | Are there signs or sharrows indicating bicycle use? |  |
| Private outdoor recreation | Is there low (pedestrian) street lights installed? |  |
| Public outdoor pay recreation | Is there high (car) street lights installed? |  |
| Public park / recreational area / gardens | Finish time |  |
| Trail / hiking / walking route |  |  |
| Pedestrian street or zone |  |  |
| Bicycle shop |  |  |
| Shopping Centers |  |  |
| How many points of confluence for formal and informal public transport along the route |  |  |
| Amenities at the points of confluence |  |  |
| What is available at the points of confluence? |  |  |
| What other transport options do you see on the route? |  |  |
| Type the other |  |  |
| What other street characteristics are present? |  |  |
| How many traffic calming? |  |  |
| How many roll-over curbs |  |  |
| Presence of street amenities |  |  |
| Do you observe pleasant hardscape features, such as fountains, sculptures, or art (public or private)? |  |  |
| Do you observe any natural bodies of water? |  |  |
| Do you observe softscape features such as gardens or landscaping? |  |  |
| Are buildings well maintained? |  |  |
| Is landscaping well maintained? |  |  |
| Is graffiti/tagging (not murals) present? |  |  |
| Rate the extent of graffiti |  |  |
| Is litter / trash present? |  |  |
| Is noticeable/excessive animal or human fouling (poop) litter present? |  |  |
| Presence of anyone walking? |  |  |
| Is there a highway/main road nearby? (is there a road entrance or can you see it) |  |  |
| Finish time |  |  |
