# Supplementary material for: Virtual Assessment of Physical Activity–Related Built Environment in Soweto, South Africa: What Is the Role of Contextual Familiarity?
Source: J Urban Health. 2024 Sep 10;101(6):1221–34. doi: 10.1007/s11524-024-00914-3 (PMC11652452; doi:10.1007/s11524-024-00914-3)
Supplement: Supplementary file 2 — Supplementary file2 (DOCX 17 KB) [file 11524_2024_914_MOESM2_ESM.docx]

**Supplementary table 2** Descriptive values of the virtual audits for MAPS-Global in Soweto, South Africa, 2023.

| Routes | Segment | | Crossing | | Route | Assessment time in minutes  Mean ± SD | | | |
| --- | --- | --- | --- | --- | --- | --- | --- | --- | --- |
|  | Num. of | Image date** (oldest – newest, mm/yy) | Number of (range) | Image date** (oldest – newest, mm/yy) | % availability of imagery | Segm. | Cross. | Route | Overall |
| **Phase 1*** | | | | | | | | | |
| Route 1 | (4-8) | 01/22 – 01/23 | (3-8) | 01/22 – 01/22 | 14.3 | 6 ± 7 | 3 ± 8 | 13 ± 6 | 22 ± 11 |
| Route 2 | (2-4) | 03/22 – 04/22 | (2-4) | 01/22 – 03/22 | 90.0 | 8 ± 13 | 3 ± 4 | 26 ± 31 | 37 ± 34 |
| Route 3 | (2-5) | 11/09 – 03/23 | (3-5) | 03/21 – 03/22 | 90.0 | 4 ± 4 | 2 ± 1 | 15 ± 15 | 20 ± 16 |
| Route 4 | (4-7) | 11/09 – 03/23 | (3-7) | 11/09 – 03/22 | 88.9 | 4 ± 3 | 2 ± 2 | 11 ± 11 | 16 ± 11 |
| Route 5 | (2-8) | 12/21 – 04/22 | (1-8) | 12/21 – 04/22 | 88.9 | 5 ± 13 | 1 ± 1 | 7 ± 4 | 13 ± 13 |
| Route 6 | (6-8) | 01/21 – 03-22 | (5-8) | 01/21– 03/22 | 88.9 | 3 ± 2 | 1 ± 1 | 5 ± 4 | 10 ± 4 |
| Route 7 | (3-6) | 01/22 – 03/22 | (2-8) | 01/22 – 03/22 | 88.9 | 4 ± 2 | 2 ± 1 | 9 ± 10 | 15 ± 10 |
| **Phase 2** | | | | | | | | | |
| Route 8 | 8 | 03/22 – 04-22 | 7 | 02/10 – 04/22 | 88.9 | 4 ± 2 | 1 ± 1 | 10 ± 8 | 16 ± 9 |
| Route 9 | 4 | 12/21 – 03/22 | 3 | 12/21 – 03/22 | 100 | 5 ± 5 | 1 ± 1 | 5 ± 3 | 11 ± 5 |
| Route 10 | 7 | 01/22 – 03/22 | 6 | 01/21 – 03/22 | 100 | 3 ± 1 | 1 ± 2 | 8 ± 7 | 12 ± 7 |

* The number of segments and crossings were not the same, hence we could not run an IRR analysis. **If the image dates coincided with the day of collection, those entries were excluded, as this was considered a mistake.
